# *APEX1* promotes the oncogenicity of hepatocellular carcinoma via regulation of *MAP2K6*

**DOI:** 10.18632/aging.204325

**Published:** 2022-10-04

**Authors:** Zhipeng Sun, Guangyang Chen, Liang Wang, Qing Sang, Guangzhong Xu, Nengwei Zhang

**Affiliations:** 1 Hepatopancreatobiliary Center, Beijing Tsinghua Changgung Hospital, School of Clinical Medicine, Institute for Precision Medicine, Tsinghua University, Beijing, China; 2 Oncology Surgery Department, Beijing Shijitan Hospital, Capital Medical University, Peking University Ninth School of Clinical Medicine, Beijing, China

**Keywords:** *APEX1*, HCC, *MAP2K6*, tumor growth

## Abstract

Objective: Apurinic/apyrimidinic endonuclease 1 (APEX1), a key enzyme responsible for DNA base excision repair, has been linked to development and progression of cancers. In this work, we aimed to explore the role of APEX1 in hepatocellular carcinoma (HCC) and elucidate its molecular mechanism.

Methods: The expression of *APEX1* in HCC tissues and matched adjacent normal tissues (*n* = 80 cases) was evaluated by immunohistochemistry. Web-based tools UALCAN and the Kaplan-Meier plotter were used to analyze the Cancer Genome Atlas database to compare expression of *APEX1* mRNA to 5-year overall survival. *APEX1* was stably silenced in two HCC cell lines, Hep 3B and Bel-7402, with shRNA technology. An *in vivo* tumorigenesis model was established by subcutaneously injecting sh-APEX1-transfected Bel-7402 cells into mice, and tumor growth was determined. We performed high-throughput transcriptome sequencing in sh-APEX1-treated HCC cells to identify the key KEGG signaling pathways induced by silencing of *APEX1*.

Results: *APEX1* was significantly upregulated and predicted poor clinical overall survival in HCC patients. Silencing *APEX1* inhibited the proliferation of HCC cells *in vivo* and *in vitro*, and it repressed invasion and migration and increased apoptosis and the percentage of cells in G1. Differentially expressed genes upon *APEX1* silencing included genes involved in TNF signaling. A positive correlation between the expression of *APEX1* and *MAP2K6* was noted, and overexpressing *MAP2K6* overcame cancer-related phenotypes associated with *APEX1* silencing.

Conclusion: *APEX1* enhances the malignant properties of HCC via *MAP2K6*. *APEX1* may represent a valuable prognostic biomarker and therapeutic target in HCC.

## INTRODUCTION

Hepatocellular carcinoma (HCC) was the most common type of primary liver cancer in adults and the sixth most commonly diagnosed cancer in 2018 [1]. Due to a limited array of treatment options, poor prognoses, and high recurrence rates, HCC has become one of the most fatal cancers in the world [2]. In recent years, however, the diagnosis and treatment of HCC have been greatly improved with methods such as surgical resection, liver transplantation, and various local treatments. Although these treatments have helped improve efficacy, the prognosis for HCC patients remains poor [3, 4]. Progress in this realm is limited by a lack of clarity regarding the exact mechanism of pathogenesis of HCC, which is known to involve multiple genetic changes.

Our present understanding of the mechanism of pathogenesis of HCC includes gene mutations, genetic changes to metabolism, abnormalities of intracellular signaling pathways, and changes to the local tumor microenvironment, with gene mutations being the most complex [5]. After certain key gene mutations occur, interactions among a series of signaling proteins are altered, potentially leading to unregulated proliferation, metastasis, and other changes of hepatocytes [6]. Because of the importance of gene mutations in mechanisms underlying cancer biology, targeted gene therapy has emerged as an attractive area of study in the field of cancer treatment in recent years. In addition, the identification of more tumor biomarkers can potentially aid in the early diagnosis of disease and serve as a method to enable monitoring of the efficacy of treatment [7–9]. Therefore, it is particularly important to identify key pathogenic genes associated with HCC.

Apurinic/apyrimidinic endonuclease 1 (APEX1) is crucial to DNA base excision repair [10, 11]. It acts via oxidation-reduction interactions and can regulate the DNA-binding activity of various transcription factors [12]. It is also known as an oxidation-reduction factor (Ref-1) and is an important multifunctional protein in the human body. APEX1 participates in various cell reactions such as cell proliferation, apoptosis, and differentiation by regulating the activity of oxidation-reduction-sensitive transcription factors [13, 14]. APEX1 is generally expressed in a variety of human cells at a high level. A study conducted by Cao L et al. showed that APEX1 is up-regulated in HCC and that this over-expression correlates with cancer aggressiveness [15]. In HCC, oxidative injury is important to the process of carcinogenesis, which promotes the development of tumors in many ways, including cell apoptosis and resistance against cell death signaling pathways [16].

Mitogen-activated protein kinase kinase 6 (MAP2K6) is an upstream kinase of the TNF signaling pathway, which is involved in various physiological and pathological processes including cell growth, development, division, and inflammatory reactions [17–19]. Guo Y et al. discovered that MAP2K6 enhanced the sensitiveness of paclitaxel for ovarian cancer via inducing autophagy [20]. At present, it is believed that MAP2K6 may be associated with the occurrence and progression of tumors and could be potentially treated as a new diagnostic or prognostic biomarker for cancers.

In this study, we determined the expression of APEX1 in human HCC tissues. Lentivirus mediated RNA interference was used to silence APEX1. The influence of APEX1 gene expression on the biological behavior of HCC cells was explored *in vivo* and *in vitro*, and we explored possible mechanisms of pathogenesis.

## MATERIALS AND METHODS

### Tissue samples

Eighty pairs of HCC/paracancerous tissues were collected from HCC patients who underwent surgical treatment in the oncology surgery department, Beijing Shijitan Hospital, Capital Medical University (Peking University Ninth School of Clinical Medicine) from August 2015 to October 2018. All patients did not receive preoperative radiotherapy or chemotherapy, and the adjacent tissues were at least 5 cm away from the edge of the tumor. All fresh tissue samples were immediately stored in a liquid nitrogen tank to protect RNA from degradation. The use of human tissues was approved by the Ethics Committee of Beijing Shijitan Hospital, Capital Medical University (NO: sjtky11-1x-2020(15)) and was conducted in accordance with the Declaration of Helsinki. We obtained written informed consent from every patient. The animal experimental protocol was approved by the Animal Care Committee of Beijing Shijitan Hospital, Capital Medical University (NO: sjtky11-1x-2019(28), sjtky11-1x-2018(108) and sjtky-1x-2019(89)).

### Cell lines and cell culture

HCC cell lines (Huh-7, SMMC-7721, Hep G2, Hep 3B, HCC-9204, Bel-7402, and Bel-7405) and the normal liver cell line L-02 were obtained from the National Biomedical Experimental Cell Resource Bank (Beijing, China). Cells were maintained in RPMI 1640 supplemented with 10% FBS (Gibco, Grand Island, NY, USA), 100 U/mL penicillin and 100 μg/mL streptomycin (Gibco, Grand Island, NY, USA). Then, the mixture was cultured in a 37°C incubator with a 5% CO_2_ environment. When the cells reached approximately 85% confluence, they were passaged at a ratio of 1:3.

### Establishment of stable *APEX1*-knockdown cells

Specific shRNA strands (F: 5′ CCG GCA GAG AAA TCT GCA TTC TAT TCT CGA GAA TAG AAT GCA GAT TTC TCT GTT TTT, R: 5′ AAT TCA AAA ACA GAG AAA TCT GCA TTC TAT TCT CGA GAA TAG AAT GCA GAT TTC TCT G) were obtained from Sangon Biotech (Shanghai, China). These shRNA were used to construct plasmid pLKO.1-puro-shRNA. A corresponding control plasmid (pLKO.1-puro-Ctrl) was also constructed. APEX1-knockdown lentiviruses were created with plasmids psPAX2 and pMD2.G (BIOFENG, Shanghai, China) and were transfected into 293T cells with pLKO.1 puro-shRNA using Lipofectamine 2000 Transfection Reagent (Thermo Fisher Scientific, MA, USA). Hep 3B and Bel-7402 cells were then infected with these lentiviruses at a multiplicity of infection of 10 and 20, respectively, and selected with puromycin (2 μg/mL) (Thermo Fisher Scientific, MA, USA). At 2 weeks post-infection, cells were harvested, and the protein expression levels of APEX1 were determined.

### Immunohistochemistry staining

All tissue samples were fixed in 10% neutral formalin, embedded in paraffin, sectioned, dewaxed with xylene and dehydrated with gradient ethanol. Then, citric acid buffer was used for high temperature antigen repair, 3% hydrogen peroxide (Solarbio, Beijing, China) was used to block endogenous peroxidase activity, and the samples were incubated at room temperature for 20 min. Next, the slides were incubated with an anti-APEX1 primary antibody (Abcam, Cambridge, UK) at 4°C overnight. The next day, the samples were incubated with an appropriate horseradish peroxidase-conjugated secondary antibody at 37°C for 30 min. After staining with 3, 3′-diaminobenzidine (Solarbio, Beijing, China), the samples were stained with hematoxylin, dehydrated with gradient ethanol, made transparent with xylene, and sealed with neutral gum. The integrated option density (IOD) of APEX1 was chosen to determine the semiquantitative protein expression. ImageJ software (version 1.2; WS Rasband, National Institute of Health, Bethesda, MD, USA) was used to conduct deconvolution and downstream analyses.

### Western blotting

Cells were collected, and total protein was extracted from cell lysates. SDS-PAGE electrophoresis was performed, and proteins were transferred electrophoretically from the gel to a polyvinylidene fluoride (PVDF) membrane. Tris-buffered saline liquid containing Tween 20 (TBST) and 5% skimmed milk powder was used to block the PVDF membrane for 2 h at room temperature. The primary anti-APEX1 antibody (Abcam, Cambridge, UK), diluted in 1% skimmed milk powder in TBST, was added, and the membrane was incubated on a shaker at 4°C overnight. The secondary antibody was added for 2 h at room temperature. Enhanced chemiluminescence reagent was added to the membrane, which was then developed in a chemiluminescence imaging system instrument.

### RT-PCR

Total RNA was extracted from cells by the Trizol method, and cDNA was synthesized according to the instructions of the manufacturer of the M-mlv reverse transcriptase kit (Takara Bio, Beijing, China). PCR amplification was performed with EvaGreen Dye (Biotium, Fremont, CA, USA) as follows: 95°C for 5 min followed by 40 cycles of 95°C for 20 s, 60°C for 30 s, and 72°C for 20 s. The primers for *APEX1* were F: 5′-GCT GCC TGG ACT CTC TCA TCA AT-3′ and R: 5′-CCT CAT CGC CTA TGC CGT AAG AA-3′. The primers for *MAP2K6* were F: 5′-TGT GCA TTT CCA TCT TGA TTC CC-3′ and R: 5′-CGC TTC TTG CCT TTC GAC TG-3′. The primers for *TNFAIP3* were F: 5′-CTG CCA GCG AGC GAG C-3′ and R: 5′-GTG CTC TCC AAC ACC TCT CC-3′. The primers for *CASP3* were F: 5′-TGG AAC CAA AGA TCA TAC ATG GAA-3′ and R: 5′-TTC CCT GAG GTT TGC TGC AT-3′. The primers for the internal control *GAPDH* were F: 5′-TGA AGG TCG GAG TCA ACG G-3′ and R: 5′-TCC TGG AAG ATG GTG ATG GG-3′.

### Cell viability measurement, colony formation assay and ethynyl deoxyuridine (EdU) assay

Cell viability was measured by the Cell Counting Kit 8 (CCK8) in accordance with the manufacturer’s instructions (Solarbio, Beijing, China). The prepared cells were collected and resuspended in 1640 medium (10% FBS) at 2 × 10^4^/mL. Then, 0.1 mL of this suspension was placed into wells of a 96-well plate and cultured for 0 h, 24 h, 48 h, or 72 h. Cell proliferation rates were quantified by measuring OD (450 nm) values with a microplate reader. For colony formation assays, treated were collected and added to a 6-well plate at a density of 1000 cells per well. The cells were cultured normally until colonies were visible. The cell colonies were stained with 0.1% crystal violet solution and photographed with a camera, then the number of cell colonies in each group was counted manually. The EdU assay was performed with an EdU kit (Roche, Indianapolis, IN, USA) according to the manufacturer’s instructions. Results were analyzed with a flow cytometer equipped with CellQuest software (BD Biosciences, San Diego, CA, USA).

### Cell migration assay (scratch wound healing assay)

HCC cells were plated and grown to 70% confluence on 6-well plates and were wounded with 1-mL pipette tips. Samples were examined at 0 and 48 h after scratching, and the wound healing status of each group was observed and photographed.

### Transwell invasion assay

Detection of cell invasion was performed in 8-μm Transwell chambers (BD Biosciences, USA). Transfected Hep 3B and Huh7 cells were suspended in serum-free medium and then seeded into the Matrigel-upper chambers at 5 × 10^4^ cells per well. DMEM containing 10% FBS (500 μL) was added into the lower chamber. After culturing for 24 h, the invasive cells were stained with 0.1% crystal violet and photographed under a light microscope. Cell numbers was counted manually.

### Flow cytometry analysis for cell apoptosis and cell cycle

Transfected Hep 3B and Huh7 cells were processed with an Apoptosis Detection Kit (Beyotime, China), according to the manufacturer’s instructions. Briefly, transfected cells were washed twice with cold PBS. After incubation with 5 μL of FITC-Annexin V and 5 μL propidium iodide for 20 min in the dark, apoptosis of Hep 3B and Huh7 was detected using a flow cytometer (FACScan; BD Biosciences, USA).

For cell cycle analyses, cells were collected and incubated with 70% ethanol at 4°C overnight for fixation. The cells were washed twice with PBS and incubated with 100 μg/mL RNase A and 50 μg/mL propidium iodide for 1 h at 37°C. The percentage of cells in each phase of the cell cycle was then measured by flow cytometry (FACScan; BD Biosciences, USA).

### Transcriptome sequencing

Total RNA was extracted from cells that had been transfected with shRNA targeting *APEX1* (sh-APEX1) or a negative control (sh-NC), and reverse transcription was performed to construct a cDNA library. High-throughput transcriptome sequencing was performed with an Illumina Hiseq 2500 system, and transcriptome sequencing data were obtained for bioinformatics analysis.

### Tumor formation in nude mice

BALB/c nude mice (male, 6–8 weeks) were adaptively raised for 2 weeks under SPF conditions, and the mice were randomly divided into two groups at the third week. There were 10 mice in each group, and each mouse was numbered. The right armpit of nude mice was subcutaneously injected with 200 μL of a 1 × 10^7^/ml cell suspension. After 8 weeks post-inoculation, the mice were killed by dislocation, and the tumor rate was calculated. The tumor tissue was completely separated, the mass of the tumor was measured, and the tumor volume was calculated using the equation: tumor volume (mm^3^) = 1/2 (long diameter × short diameter × short diameter). For the Establishment of the orthotopic liver cancer model, the mice were anesthetized with 3% to 5% isoflurane, and the left lobe of the liver was exposed along the midline of the upper abdomen. Suspended cells are sucked into the syringe, punctured into the left lobe of the liver about 5 mm at an angle of 30°, and the cells are slowly injected into the liver tissue and then multi-point injections are performed. Then, gently press the injection site with a cotton ball for about 1 minute to reduce bleeding and cell suspension leakage. Finally, the peritoneum was closed with 4–0 sutures intermittently, and the skin incision was closed with suture nails. After execution, liver tissue was taken for analysis of tumor formation. The animal experimental protocol was approved by the Animal Care Committee of Beijing Shijitan Hospital, Capital Medical University (NO: sjtky11-1x-2019(28), sjtky11-1x-2018(108) and sjtky-1x-2019(89)).

### Statistical analysis

All statistical analyses were performed in Statistical Product and Service Solutions (SPSS) 20.0 software (SPSS, Chicago, IL, USA). SigmaPlot12.3 (Systat Software, San Jose, CA, USA) and GraphPad Prism 5.0 (GraphPad Software, La Jolla, CA, USA) software were used to draw graphs. Student’s *t*-test, one-way analysis of variance (ANOVA), and a rank-sum test were flexibly applied according to the conditions at hand. Differences for which *P* < 0.05 were regarded as statistically significant.

## RESULTS

### *APEX1* is up-regulated in HCC

**Figure 1:**
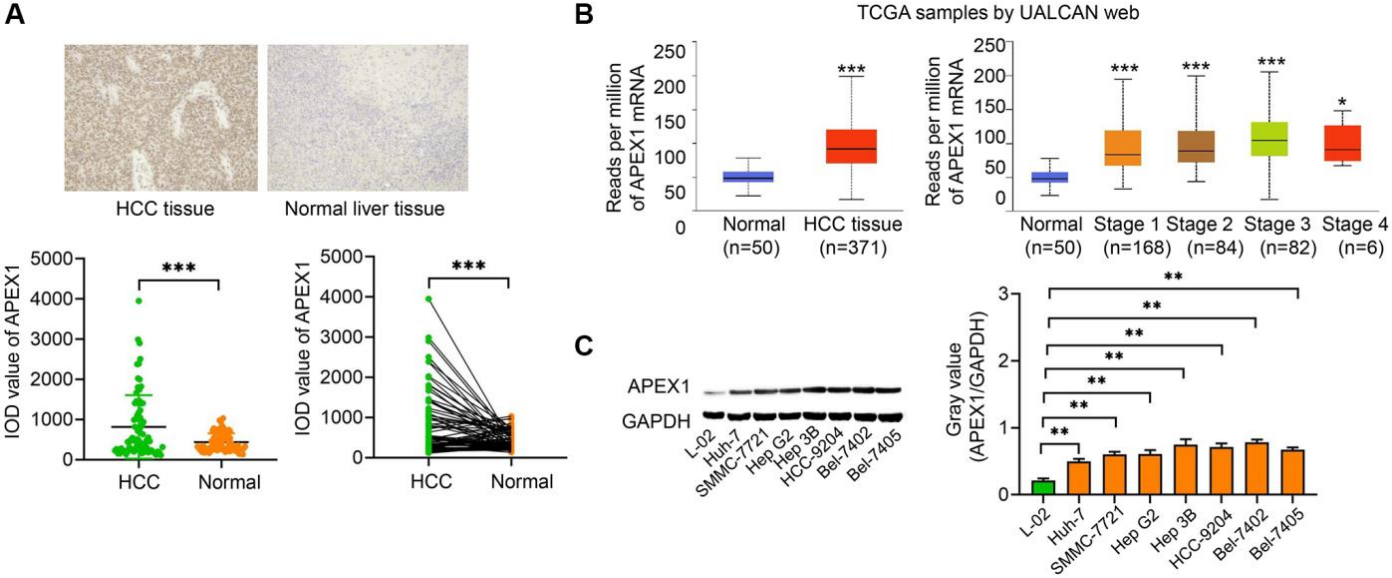
***APEX1* is a novel HCC-related oncogene.** (**A**) The expression of *APEX1* was evaluated by immunohistochemistry analysis of HCC specimens and matched adjacent normal tissues (*n* = 80 cases). (**B**) Bioinformatics analysis using the UALCAN tool on TCGA dataset compared the mRNA expression of *APEX1* in HCC (*n* = 371) and normal (*n* = 50) tissues (Left). The expression of *APEX1* was also analyzed in HCC tissues at various stages (Right). (**C**) APEX1 protein expression was evaluated in HCC cells and normal hepatic cells by a Western blot assay. ^*^P < 0.05, ^**^P < 0.01, ^***^P < 0.001.

Immunohistochemistry was used to measure the expression of APEX1 in HCC tissues and adjacent normal tissues (*n* = 80). As shown in Figure 1A, expression of APEX1 protein in HCC tissues was higher relative to that in adjacent normal tissues. Furthermore, we measured the expression of *APEX1* mRNA using UALCAN, an online server derived from the Cancer Genome Atlas (TCGA) dataset [17]. The results indicated that expression of *APEX1* mRNA in HCC tissues at multiple stages was higher than that in normal tissues (Figure 1B). Western blotting was used to measure the expression of APEX1 protein in HCC cell lines (Huh-7, SMMC-7721, Hep G2, Hep 3B, HCC-9204, Bel-7402, and Bel-7405) and in the normal liver cell line L-02. As shown in Figure 1C, APEX1 expression in the HCC cell lines was higher, to varying degrees, than was expression in L-02.

### Relationship of *APEX1* expression and clinical pathologic characteristics of HCC

**Figure 2:**
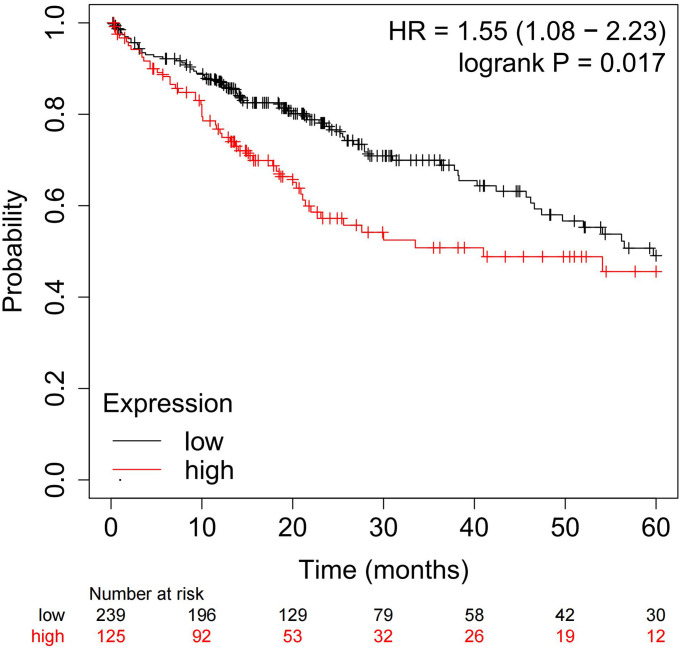
Identification of the correlation between 5-year overall survival of HCC patients and *APEX1* expression by applying the Kaplan-Meier plotter on TCGA dataset.

The relationships between APEX1 expression and clinical characteristics of HCC patients are shown in Table 1. Statistical analyses suggested that APEX1 expression level increased with increasing pathological grade and TNM stage of HCC (*P < 0.05*). Moreover, the Kaplan-Meier plotter [18], a web-based tool that uses TCGA databases to assess the effect of changes to the expression of 54,000 genes (at the level of mRNA, miRNA, or protein) on survival, was used. This plotter allows the prediction of the prognostic value of the tumor-specific expression of various genes. When we analyzed the correlation of *APEX1* expression with HCC patient survival in this way, the results suggested that patients with high *APEX1* expression exhibited a lower 5-year survival rate compared with those with low *APEX1* expression (Figure 2).

**Table 1:** Relevance between *APEX1* expression and clinical pathologic characteristics of HCC.

**Variable**	**No. of patients *N* (%)**	**APEX1 expression**		**χ^2^**	***P***
		**High (*n* = 31) *N* (%)**	**Low (*n* = 49) *N* (%)**		
Gender					
Male	64 (0.8)	25 (0.313)	39 (0.487)	0.013	0.908
Female	16 (0.2)	6 (0.075)	10 (0.125)		
Age (years)					
≤50	20 (0.25)	7 (0.088)	13 (0.162)	0.158	0.691
>50	60 (0.75)	24 (0.3)	36 (0.45)		
TNM stage					
I~II	39 (0.488)	10 (0.125)	29 (0.362)	5.509	0.019
III~IV	41 (0.512)	21 (0.263)	20 (0.25)		
Pathological grading					
Well (I)	2 (0.025)	0 (0)	2 (0.025)	7.167	0.027
Moderate (II–III)	67 (0.838)	23 (0.288)	44 (0.55)		
Poor (IV)	11 (0.137)	8 (0.1)	3 (0.037)		

### Downregulation of *APEX1* inhibited HCC cell growth

**Figure 3:**
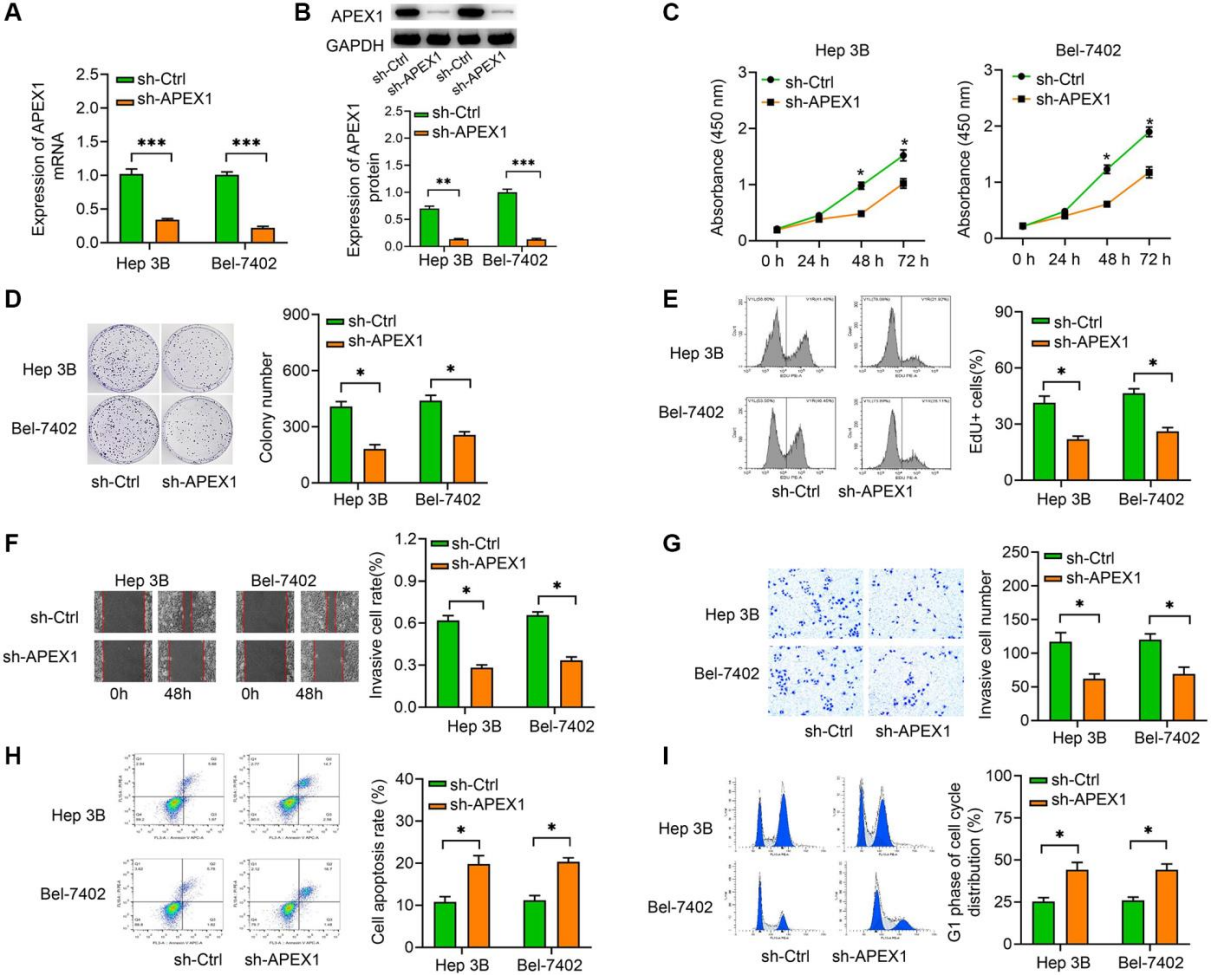
***APEX1* modulates HCC cell proliferation, migration, invasion, apoptosis and cell cycle distribution *in vitro*.** (**A**, **B**) The efficiency of transfection of sh-APEX1 was detected by qRT-PCR and Western blot assays. (**C**–**E**) CCK-8 (**C**), colony formation (**D**) and EdU assays (**E**) were performed to evaluate the effect of *APEX1* on the proliferation of HCC cells. (**F**, **G**) Wound healing (**F**) and Transwell assays (**G**) were performed to evaluate the migration and invasion abilities of HCC cells. (**H**, **I**) Flow cytometry was performed to evaluate apoptosis (**H**) and cell cycle distribution (**I**). ^*^P < 0.05 vs. sh-Ctrl.

To identify functional roles of APEX1 protein in HCC cells, we silenced the *APEX1* gene in the two HCC cell lines, Hep 3B and Bel-7402, that were determined to have the highest expression of APEX1. The results of qRT-PCR and Western blot analyses suggested that the expression of *APEX1* in the sh-APEX1 group was markedly lower than in the sh-NC group (Figure 3A, 3B). We next evaluated the vitality of these two cell lines by performing CCK-8 (Figure 3C), colony-forming (Figure 3D) and EdU assays (Figure 3E). These results indicated that *APEX1* knockdown suppressed the proliferation of HCC cells.

We also examined the effects of *APEX1* on the invasion and migration of HCC cells. Inhibition of *APEX1* expression appeared to decrease the invasiveness of the HCC cells (Figure 3F). Similarly, *APEX1* knockdown attenuated the migration ability of HCC cells (Figure 3G). Moreover, we applied flow cytometry to detect whether the anti-proliferation activity of *APEX1* is associated with cell apoptosis or the cell cycle. The results showed a significant increase in cell apoptosis upon *APEX1* knockdown (Figure 3H). Analysis of the cell cycle distribution revealed that the percentage of cells in G1 was increased upon the inhibition of *APEX1* in both cell lines (Figure 3I). Taken together, these data indicated that *APEX1* promotes HCC cell proliferation, invasion, and migration, and it inhibits cell apoptosis and alters cell cycle distribution of HCC cells.

### *APEX1* promotes tumor growth *in vivo*

**Figure 4:**
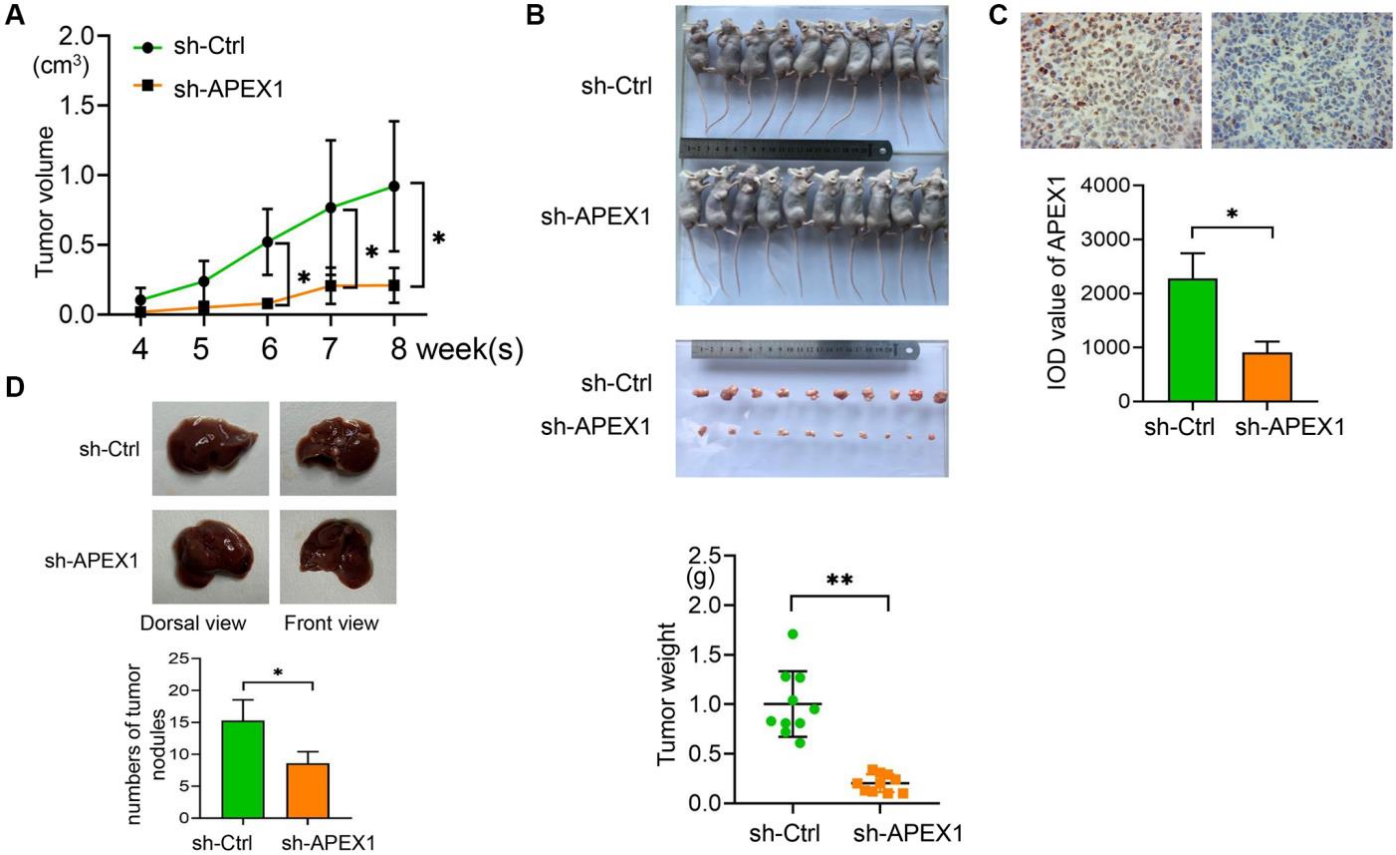
***APEX1* promotes tumor growth *in vivo*.** (**A**) An *in vivo* tumorigenesis model was established, and tumor volumes were measured every 7 days. (**B**) Tumors were exercised from mice and photographed. Tumor weight was determined. (**C**) *APEX1* expression in isolated tumors was evaluated by immunohistochemistry. (**D**) The establishment of the orthotopic liver cancer model and numbers of tumor nodules was evaluated. ^*^P < 0.05.

To investigate the role of *APEX1* in HCC tumor growth *in vivo*, we established a tumorigenesis model by subcutaneously injecting the sh-APEX1-transfected HCC cell line Bel-7402 into a nude mouse model. Tumor volume was monitored every week, and mice were sacrificed 8 weeks after inoculation for the determination of tumor weight. Compared with the control group, implantation of cells under-expressing *APEX1* caused a significantly increased tumor growth in terms of tumor volume and weight (Figure 4A, 4B). Results of immunohistochemical analyses of isolated tumors confirmed a decreased expression of APEX1 protein in the sh-APEX1 group (Figure 4C). In addition, the establishment of the orthotopic liver cancer model revealed that APEX1 knockdown suppressed numbers of tumor nodules (Figure 4D). These findings indicate that silencing of APEX1 can reduce the tumorigenicity of HCC cells *in vivo*, thereby potentially inhibiting the development of HCC.

### Transcriptome analysis of sh-APEX1-treated HCC cells

**Figure 5:**
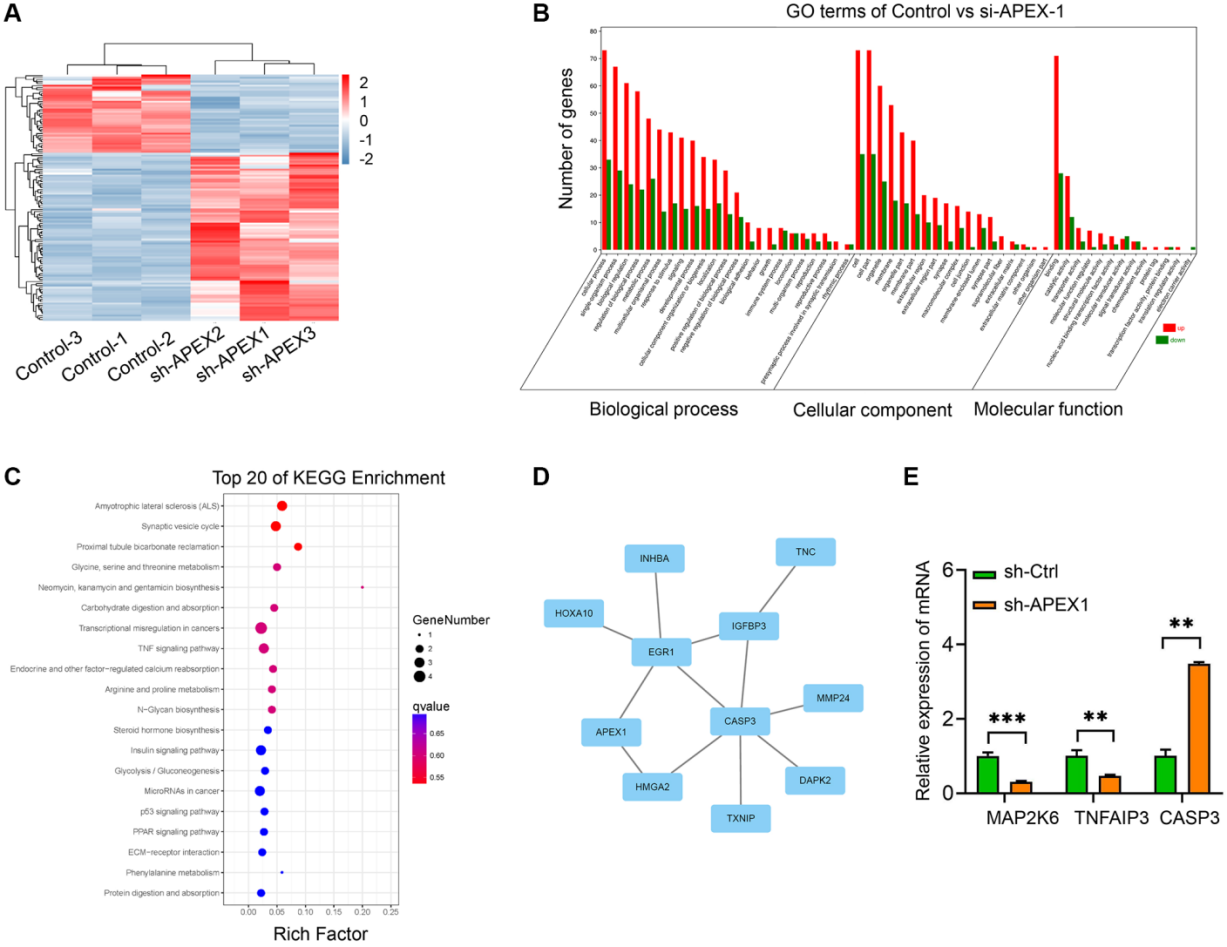
**Transcriptome analysis of sh-APEX1-treated HCC cells.** (**A**) The heat map of DEGs between sh-APEX1 and sh-Ctrl HCC cells. (**B**, **C**) GO (**B**) and KEGG signaling pathway (**C**) analyses of DEGs. (**D**) Protein interaction network analysis of DGEs. (**E**) Expression of *MAP2K6*, *TNFAIP3*, and *CASP3*, which are enriched in the TNF signaling pathway, were confirmed by qRT-PCR. ^**^P < 0.01.

In order to validate the transcriptome changes in sh-APEX1-transfected HCC cells, we performed high-throughput transcriptome sequencing. The stratified cluster heat map demonstrated the distribution of differential genes between the sh-APEX1 group and the control group. There were 84 up-regulated genes and 39 down-regulated genes in the sh-APEX1-treated HCC cells (Figure 5A).

A total of 123 differentially expressed genes (DEGs) were subjected to Gene Ontology (GO) enrichment and Kyoto Encyclopedia of Genes and Genomes (KEGG) signaling pathway analysis. The molecular functions that the DEGs were found to be involved in included binding, catalytic activity, transporter activity, molecular function regulation, structural molecule activity, nucleic acid binding transcription factor activity and molecular transducer activity. The cellular components of the gene products included cell, cell part, organelle, membrane, organelle part, membrane part, extracellular region, extracellular region part, and synapse. The biological processes involved included cellular process, single-organism process, biological regulation, regulation of biological process, metabolic process, multicellular organismal process, and response to stimulus and signaling (Figure 5B).

KEGG enrichment analysis showed that after silencing of *APEX1*, the top 20 signaling pathways that DEGs are involved in were related to amyotrophic lateral sclerosis (ALS); the synaptic vesicle cycle; proximal tubule bicarbonate reclamation; glycine, serine and threonine metabolism; neomycin, kanamycin and gentamicin biosynthesis; carbohydrate digestion and absorption; transcriptional misregulation in cancers; the tumor necrosis factor (TNF) signaling pathway; endocrine and other factor-regulated calcium reabsorption and arginine and proline metabolism (Figure 5C). Results of a differential gene protein interaction network analysis showed that *APEX1*, *CASP3*, *EGR1*, *IGFBP3*, *INHBA*, *TNC*, *HOXA10*, *IGFBP3*, *MMP23*, *HMGA2*, *DAPK2* and *TXNIP* genes made up the core protein interaction network (Figure 5D).

We next performed a qRT-PCR analysis to evaluate the expression of 3 DGEs that are enriched in the TNF signaling pathway: those encoding mitogen-activated protein kinase kinase 6 (*MAP2K6*), TNF alpha-induced protein 3 (*TNFAIP3*) and caspase 3 (*CASP3*). According to this assay, the expression of MAP2K6 and TNFAIP3 were evidently decreased, while CASP3 expression was significantly increased, in sh-APEX1-treated HCC cells as compared to control-treated cells (Figure 5E).

### Enhancing of *MAP2K6* attenuates the anti-oncogenic function of silenced *APEX1*

**Figure 6:**
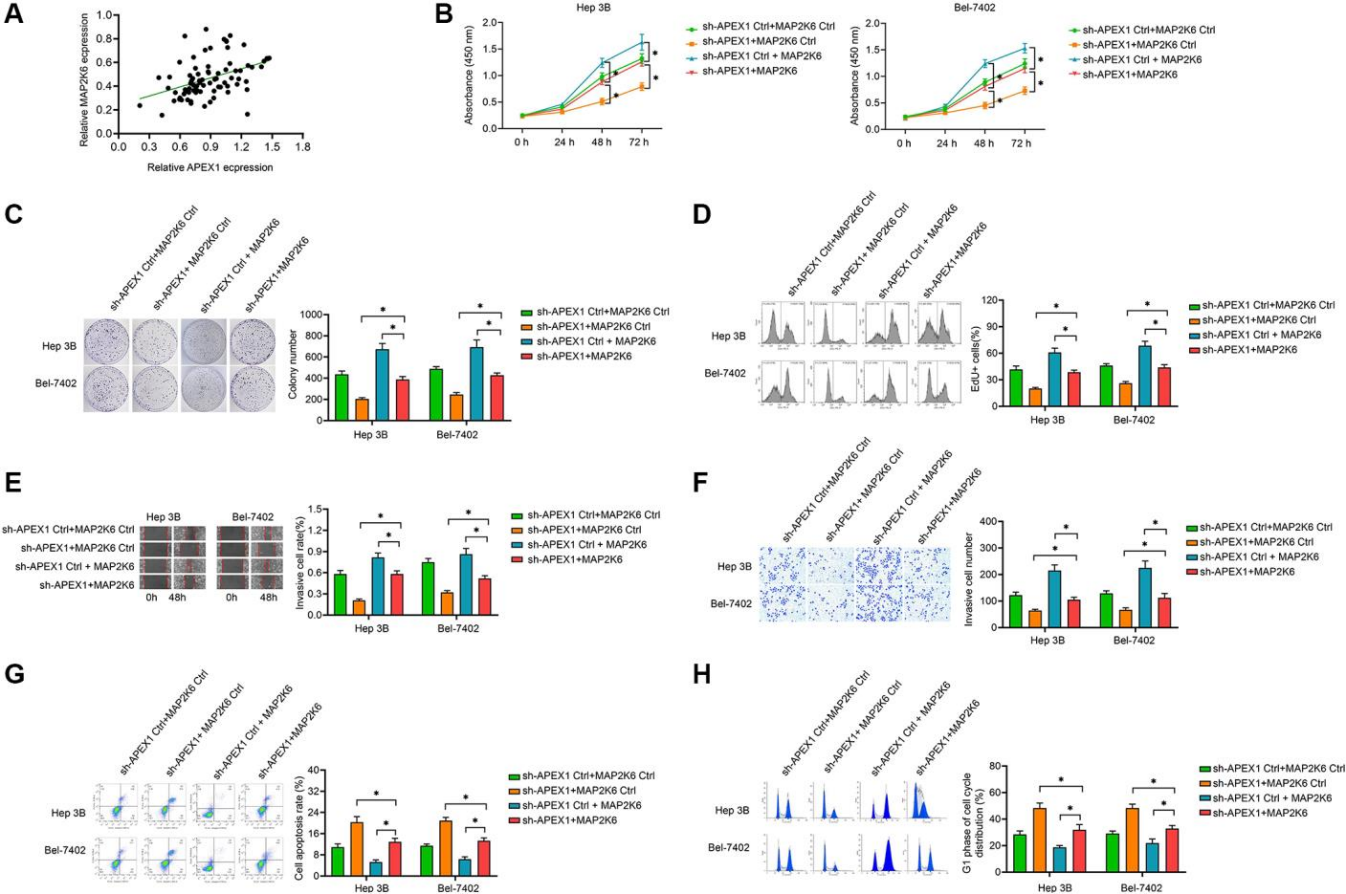
**Enhancing of the expression of *MAP2K6* attenuates the anti-oncogenic effect of silencing of *APEX1*.** (**A**) The positive correlation between *APEX1* expression and *MAP2K6* expression in HCC tissues. (**B**–**D**) Cell vitality was evaluated with CCK-8 (**B**), colony formation (**C**) and EdU assay (**D**) assays in HCC cells. (**E**, **F**) Wound healing (**E**) and Transwell (**F**) assays were performed to evaluate the migration and invasion abilities of the HCC cells. (**G**, **H**) Flow cytometry was performed to evaluate apoptosis (**G**) and cell cycle distribution (**H**). ^*^P < 0.05.

Based on the down-regulation of *MAP2K6* in sh-APEX1-treated HCC cells and a positive expression correlation between *APEX1* and *MAP2K6* in HCC tissues (Figure 6A), we predicted that *APEX1* enhances the malignant properties of HCC via its impact on *MAP2K6* expression. We therefore sought to investigate whether *MAP2K6* was responsible for the declined proliferation, invasion, and migration, enhanced cell apoptosis and altered cell cycle distribution induced by silencing of *APEX1*. We enhanced the expression of *MAP2K6* in sh-APEX1-treated HCC cells and found that overexpression of *MAP2K6* could abolish the role of inhibition of *APEX1* in attenuating cell vitality, colony formation and proliferation (Figure 6B–6D). The decreases in cell invasion and migration induced by sh-APEX1 were also reversed by *MAP2K6* overexpression (Figure 6E, 6F). In addition, the significant increase in cell apoptosis and cells in the G1 phase caused by *APEX1* knockdown were also attenuated by *MAP2K6* expression (Figure 6G, 6H). These data indicate that *MAP2K6* can inhibit the anti-tumorigenic function of sh-APEX1 in HCC cells.

## DISCUSSION

Hepatocellular carcinoma is the most common type of primary liver cancer in adults [1, 2]. Although exact details of its mechanism of pathogenesis are still unclear, the malignant transformation of liver cells involves multiple genetic aberrations. At present, the main mechanisms of pathogenesis are thought to include gene mutations as well as changes to metabolism, intracellular signaling pathways and the local tumor microenvironment [19]. After some key gene mutations, a cascade of changes to signaling proteins occurs, and these changes then lead to the infinite proliferation and other behavioral changes. With the rapid development of genome sequencing technology in recent years, it has been found that some common gene mutations in liver cancer can also cause changes in other cells types. Increasing attention has thus been devoted toward targeted gene therapy as a method of cancer treatment [20]. At the same time, tumor markers can aid in effective early diagnosis of disease, and can also be a tool for monitoring responses to therapy. Therefore, it is particularly important to identify key pathogenic genes [21, 22].

APEX1 is a multifunctional protein that is widely expressed in various types of human cells. It is encoded by an important gene [10, 12]. APEX1 has two key roles. It is not only responsible for repairing the apurinic/apyrimidinic (AP) sites generated by various factors and thus maintaining the stability of genomic DNA, but it is also responsible for regulating DNA binding activity by controlling the activation state of transcription factors [12–14]. Whereas the genomic instability that results from improperly repair AP sites typically results in controlled cellular self-destruction, in the presence of abnormal binding of transcription factors to DNA, the cell may undergo malignant transformation and develop into a tumor.

It has been shown that there are significantly higher levels of APEX1 protein in tumor cells compared to normal cells [15, 23, 24]. In addition, Di Maso et al. reported that the expression of *APEX1* mRNA was significantly increased in HCC tissues [25]. In our study, we measured the expression of APEX1 protein and mRNA in HCC tissues and cells and in normal liver tissues and cells. Indeed, we found that expression of the *APEX1* gene was significantly up-regulated in HCC. Moreover, *APEX1* expression was found to be closely associated with the pathological grade of HCC: highly expressed *APEX1* predicted poor clinical overall survival in HCC patients. This indicates that *APEX1* is up-regulated in HCC and that it may contribute to the development of HCC.

Aberrant expression of *APEX1* has been frequently identified in cancers and plays crucial roles in the modulation of multiple oncogenic properties. For example, *APEX1* expression is inversely associated with survival among patients with breast cancer [26], gastric cancer [27] and prostate cancer [28]. In these studies, *APEX1* was shown to be essential in modulating the growth of the malignancies. These findings are consistent with those reported here and demonstrate that *APEX1* plays a crucial role in the tumorigenesis of multiple cancers. The knockdown of *APEX1* repressed proliferation, invasion, and migration, accelerated cell apoptosis, and the percentage of cells in the G1 phase of the cell cycle of HCC-derived cells. To further verify those effects, we performed a tumor study in nude mice. We found that silencing of *APEX1* markedly reduced the body weight and tumor volume of nude mice, which indicated that sh-APEX1 could reduce the tumorigenic characteristics of HCC cells.

Next, to explore the molecular mechanism that would explain the role of *APEX1* in the development of HCC, we screened for differentially expressed genes in HCC cells with knocked down *APEX1* expression. We then identified the KEGG signaling pathways induced by the silencing of *APEX1*. KEGG [29] enrichment analysis indicated that DEGs were mainly involved in amyotrophic lateral sclerosis, the synaptic vesicle cycle, proximal tubule bicarbonate reclamation, glycine biosynthesis, and the TNF signaling pathway. Three TNF pathway-related DEGs, *MAP2K6*, *TNFAIP3* and *CASP3*, were selected for analysis, due to their potential role as downstream regulatory genes and signaling pathways related to *APEX1*. We found by qRT-PCR analysis that *TNFAIP3* and *MAP2K6* were down-regulated and *CASP3* was up-regulated after silencing of *APEX1*.

*MAP2K6* plays an important role in the p38 MAP kinase signaling cascade, which regulates many stress-induced responses, is related to multiple pathological conditions and plays a role in many cellular processes, including differentiation, in bone, muscle, and adipose tissue [30, 31]. Based on the down-regulation of *MAP2K6* in sh-APEX1-treated HCC cells and a positive correlation of expression of *APEX1* and *MAP2K6* in HCC tissues, we suggest that *APEX1* enhances the malignant properties of HCC via *MAP2K6*. Thus, we enhanced the expression of *MAP2K6* in *APEX1*-silenced HCC cells to evaluate the anti-oncogenic function of *MAP2K6* with silenced *APEX1*. Here, we found that forced expression of *MAP2K6* could abolish the role of inhibition of *APEX1* in attenuating cell vitality, colony formation, proliferation, migration and invasion. In addition, a significant increase in the cell apoptosis and cells in the G1 phase that was caused by *APEX1* knockdown were also attenuated by *MAP2K6* overexpression. These data indicate that *MAP2K6* can inhibit the anti-tumorigenic function of sh-APEX1 in HCC cells. The above evidence enhanced our understanding of the molecular mechanisms of the role of *APEX1* in regulating the aggressiveness of HCC.

## CONCLUSION

We revealed that *APEX1* is an independent prognostic factor that promotes HCC growth and metastasis through its interaction with *MAP2K6*. We confirmed that the upregulation of *APEX1* is a common phenomenon in HCC tissues and cell lines and is significantly correlated with the pathological grade and TNM stage of HCC. In addition, the novel HCC-related gene *APEX1* enhances the malignant properties of HCC via the overexpression of *MAP2K6*. *APEX1* may represent a valuable prognostic biomarker and therapeutic target for HCC.
